# Supplementary material for: Investigating associations between blood metabolites, later life brain imaging measures, and genetic risk for Alzheimer’s disease
Source: Alzheimers Res Ther. 2023 Feb 22;15:38. doi: 10.1186/s13195-023-01184-y (PMC9945600; doi:10.1186/s13195-023-01184-y)
Supplement: Supplementary file 6 — Additional file 6: Supplementary file 6: Figure 1. A. Plot showing the Zsummary preservation statistics for each module against the module size. Thresholds for moderate and strong evidence of module preservation are indicated. B. Plot showing the median rank for module preservation. Modules are ranked from most preserved (lowest number) to least preserved (highest number). [file 13195_2023_1184_MOESM6_ESM.pdf]

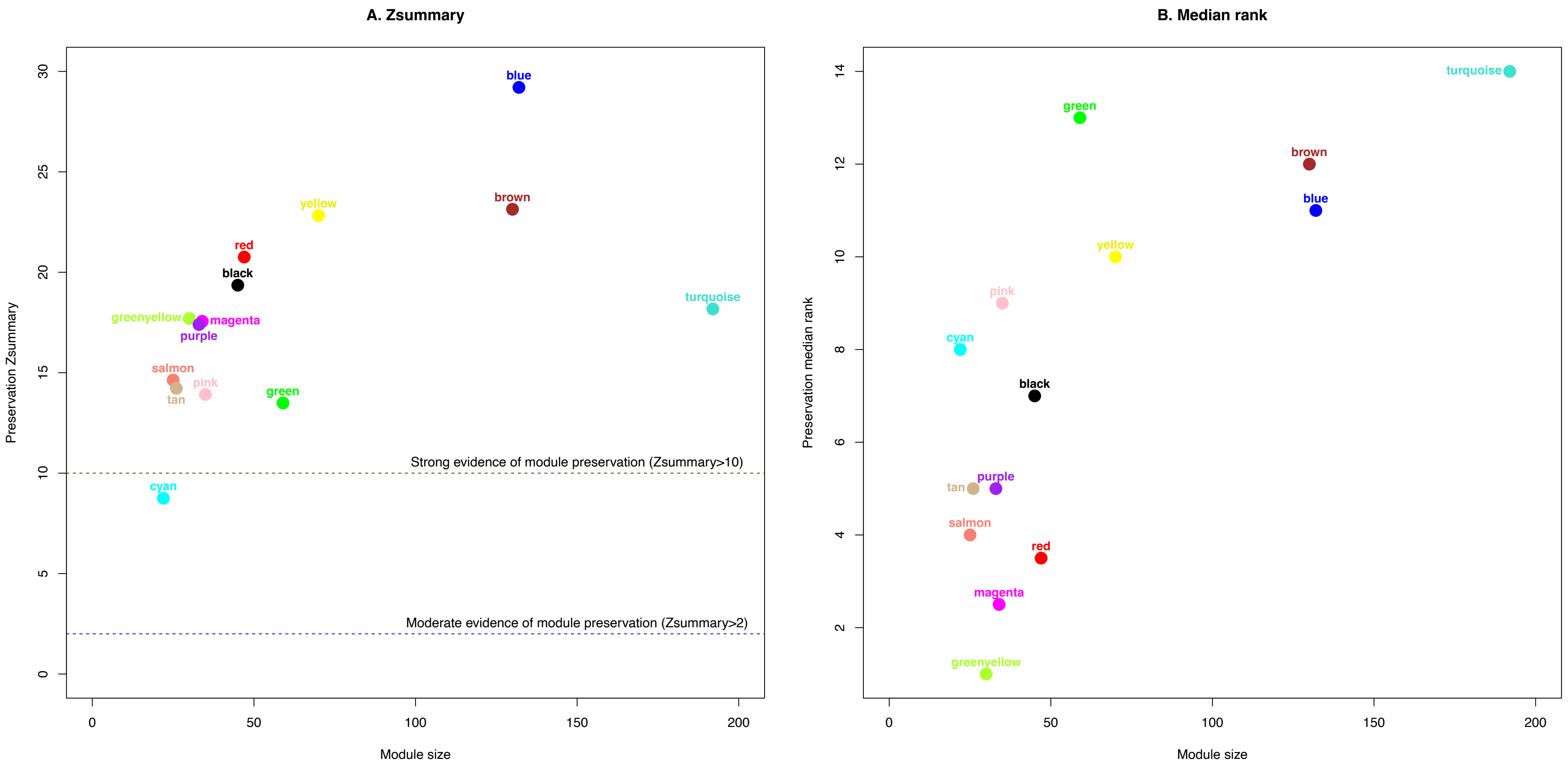

Supplementary Figure 1. A. Plot showing the Zsummary preservation statistics for each module against the module size. Thresholds for moderate and strong evidence of module preservation are indicated. B. Plot showing the median rank for module preservation. Modules are ranked from most preserved (lowest number) to least preserved (highest number).
